# An EPID-based method for comprehensive verification of gantry, EPID and the MLC carriage positional accuracy in Varian linacs during arc treatments

**DOI:** 10.1186/s13014-014-0249-8

**Published:** 2014-11-26

**Authors:** Pejman Rowshanfarzad, Conor K McGarry, Michael P Barnes, Mahsheed Sabet, Martin A Ebert

**Affiliations:** School of Physics, University of Western Australia, Crawley, WA 6009 Australia; Radiotherapy Physics, Northern Ireland Cancer Centre, Belfast Health and Social Care Trust, Belfast, UK; Centre for Cancer Research and Cell Biology, Queen’s University Belfast, Belfast, UK; Department of Radiation Oncology, Calvary Mater Newcastle Hospital, Newcastle, NSW 2310 Australia; Department of Radiation Oncology, Sir Charles Gairdner Hospital, Nedlands, WA 6009 Australia

**Keywords:** Linac, Varian, Sag, Gantry, EPID, MLC, Collimator, Quality assurance, Arc

## Abstract

**Background:**

In modern radiotherapy, it is crucial to monitor the performance of all linac components including gantry, collimation system and electronic portal imaging device (EPID) during arc deliveries. In this study, a simple EPID-based measurement method has been introduced in conjunction with an algorithm to investigate the stability of these systems during arc treatments with the aim of ensuring the accuracy of linac mechanical performance.

**Methods:**

The Varian EPID sag, gantry sag, changes in source-to-detector distance (SDD), EPID and collimator skewness, EPID tilt, and the sag in MLC carriages as a result of linac rotation were separately investigated by acquisition of EPID images of a simple phantom comprised of 5 ball-bearings during arc delivery. A fast and robust software package was developed for automated analysis of image data. Twelve Varian linacs of different models were investigated.

**Results:**

The average EPID sag was within 1 mm for all tested linacs. All machines showed less than 1 mm gantry sag. Changes in SDD values were within 1.7 mm except for three linacs of one centre which were within 9 mm. Values of EPID skewness and tilt were negligible in all tested linacs. The maximum sag in MLC leaf bank assemblies was around 1 mm. The EPID sag showed a considerable improvement in TrueBeam linacs.

**Conclusion:**

The methodology and software developed in this study provide a simple tool for effective investigation of the behaviour of linac components with gantry rotation. It is reproducible and accurate and can be easily performed as a routine test in clinics.

## Background

Volumetric Modulated Radiation Therapy (VMAT) has proved to produce superior outcomes in many treatment sites [1-3]. VMAT is faster, more accurate, and more cost effective compared to its predecessors such as conformal radiation therapy and IMRT. Due to the increased complexity of arc methods, it is a requirement to perform more vigorous tests on mechanical performance of the multi-ton gantry, field-limiting collimators, and the imaging systems mounted on the body of the machine.

The heavy components in the gantry head are affected by gravity during arc delivery; therefore the gantry does not follow a perfect circular pattern around its axis. Imperfections in collimator alignment during arc deliveries (due to gravity) in addition to the eccentricity of gantry rotation result in uncertainties in field shapes with respect to planned treatment fields, since these are not considered in the planning systems.

Electronic portal imaging devices (EPIDs) have been widely used for patient positioning, quality assurance tests on linear accelerators, and pre- and post-treatment dosimetry verification [4-7]. They are routinely tested for geometric accuracy, image quality, and operational safety [8,9]. However, gantry rotation during arc deliveries can affect the accuracy of EPID positioning, since the detector is mounted on a supporting arm with fasteners and gear belts. In addition, the arm has joints which are not quite rigid. This could degrade image quality and affect the accuracy of EPID-based mechanical and dosimetric QA.

There have been studies in the literature on the mechanical behaviour of gantry or collimators or EPIDs during arc deliveries. Those have been discussed in detail in previous papers [10-13]. In this study an EPID-based methodology in conjunction with a simple designed phantom and robust software have been used to investigate the sag in gantry, collimator assembly, and EPID, in addition to the tilt and skewness of EPID detector panels during arcs. The phantom and algorithms used in this work are modified versions of the ones explained in a previous paper [13]. Twelve Varian linacs of different models including Trilogy, Clinac iX, and TrueBeam clinically used in different radiotherapy centres have been investigated and discrepancies are statistically compared.

## Methods

### Devices

Twelve Varian linear accelerators (Varian Medical Systems, Palo Alto, CA) including Trilogy (3), Clinac iX (6), and TrueBeam (3) models were used for irradiations. All tested linacs were operated in the 6 MV photon mode. Linac specifications are given in Table 1. They were all equipped with Millennium™ 120 leaf MLC with two banks (left and right) each with 60 tungsten alloy rounded-end leaves mounted on a carriage. The 40 central leaves in each bank are 0.5 cm thick (at the isocentre level) and are called inner leaves. The peripheral leaves which are called the outer leaves are 1.0 cm thick (except for the first and last leaf pairs which are 1.4 cm thick). Each leaf is equipped with a motor and encoder, and is driven by the MLC controller which drives the leaf along the carriage. Each of the MLC carriage assemblies weighs about 36 kg.

**Table 1:** **Specifications of the linear accelerators used in this study**

**Linac number**	**Linac model**	**Detector model**	**Years in service**
1	Varian-TrueBeam	aS1000	0.5
2	Varian-TrueBeam	aS1000	1.5
3	Varian-TrueBeam	aS1000	1
4	Varian-Trilogy	aS1000	2
5	Varian-Clinac iX	aS1000	3
6	Varian-Trilogy	aS1000	5
7	Varian-Clinac iX	aS500	5
8	Varian- Clinac iX	aS500	7
9	Varian- Clinac iX	aS500	7
10	Varian-Clinac iX	aS500	7.5
11	Varian-Clinac iX	aS1000	7.5
12	Varian-Trilogy	aS1000	7.5

The linacs used in this study were equipped with either of the aS500 or aS1000 PortalVision image detectors attached to the machine by ExactArm™ type supporting arms. These imagers weigh about 7 kg. The active areas of the detector panels were 40 × 30 cm^2^ containing 512 × 384 pixels in aS500 EPIDs and 1024 × 768 pixels in the aS1000 model.

### Test procedure

Megavoltage EPID images were acquired in DICOM format and were automatically dark-field and flood-field corrected by the imaging system software.

To test the Trilogy, Clinac iX linacs, EPID images were acquired using 360 MU irradiations at a nominal rate of 600 MU/min in continuous (cine) image acquisition mode at a rate of 7.5 frames per second (6 frames per image). The EPID was positioned at 150 cm source-to-detector distance (SDD) during 360° gantry rotations, which yielded one image per ~4° rotation. Images were taken for 18 × 18 cm^2^ MLC-defined fields. For TrueBeam linacs, cine EPID images could not be saved since the developer mode was not available on them; therefore, images were acquired in integrated mode with 20 MU irradiations per image in 10 degree intervals, providing 37 images for an entire gantry rotation.

Each set of measurements were performed three times to check the reproducibility of results. The tests were performed in both clockwise (CW) and counter-clockwise (CCW) directions, and at 0 and 90 degree collimator angles to assess any possible interplay effects. The data acquired at zero degree gantry angle were taken as reference to determine relative deviations at other angles, since reference machine data acquisition and calibrations are performed at zero gantry angle. All results were scaled back to the isocentre plane, except for the changes in SDD during arc.

All gantry angles are stated according to the IEC convention [14]. Data analysis and algorithm development were carried out using the MATLAB programming language and software (The Mathworks Inc., Natick, MA).

**Figure 1:**
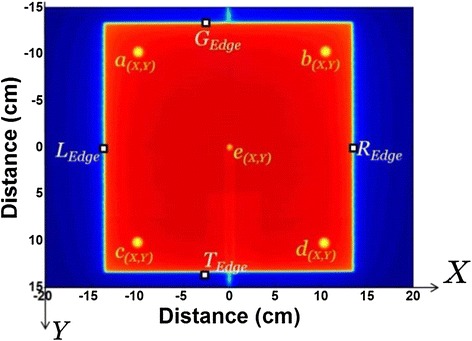
**A sample image acquired for characterization of Varian linac components at SDD = 150 cm.** The shadows of the markers attached to the head are labelled as (a), (b), (c) and (d). The ball bearing at the isocentre is named (e). The X and Y directions represent the cross-plane and in-plane directions, respectively.

The phantom used in this study includes five 4.8 mm diameter tungsten carbide ball bearings, four of them rigidly attached to the gantry head, and the fifth attached to the end of a Perspex rod. The rod was fixed to the treatment table top and the ball bearing was positioned at the nominal linac isocentre using the room lasers while the collimator and couch were set at zero angle. A sample snapshot is shown in Figure 1.

A single set of images acquired during an entire gantry rotation provides data for all of the components under investigation, and the software needs to be run only once to load all images and output the whole set of results. The basis of the algorithm for determination of the centre of each ball bearing and the field edges has been explained elsewhere [12,13,15]. In this study, a new version of the algorithm was developed to detect five ball bearings, and conservative smoothing technique was added to the method to remove noise spikes without losing high spatial frequency details. A rank filtering mask (2 × 2) rank 4 was used on all images. The algorithm was also modified to use the extra information from the new phantom settings, to extract the EPID tilt and skewness.

The overall time estimate for the procedure is about 9 minutes for Trilogy and Clinac iX linacs, including ~6 minutes for the setup, less than 2 minutes for image acquisition and export, and ~1 minute for data processing on a computer with 2.60 GHz CPU and 4.00 GB RAM. The image acquisition time was about 2 hours for the TrueBeam machines, due to the need to take images in integrated mode.

### Analysis methods

#### EPID sag

The position of the centre of ball bearing (*e*) fixed at the isocentre in each image is compared with the reference image acquired at zero gantry angle based on Equations (1) and (2).

1 $$ {\left( EPID\ Sa{g}_X\right)}^{\theta }=\left[{e}_X^{\theta }-A \cos \theta \right]-{e}_X^0 $$

2 $$ {\left( EPID\ Sa{g}_Y\right)}^{\theta }=\left[{e}_Y^{\theta }-B \sin \theta \right]-{e}_Y^0 $$

where (*EPID Sag*_*X*_)^*θ*^ and (*EPID Sag*_*Y*_)^*θ*^ are the EPID sag in X and Y (cross-plane and in-plane) directions at θ gantry angle; ${e}_X^{\theta }$ and ${e}_Y^{\theta }$ are the positions of the centre of ball bearing (*e*) in X and Y directions at θ gantry angle; the terms (*A* cos *θ*) and (*B* sin *θ*) are applied to correct for the lateral and vertical displacements induced as a result of misalignments of lasers or displacement of ball bearing (*e*) from the isocentre. These misalignments introduce a simple periodic function into the geometric location of the marker image on the EPID during a whole gantry rotation, since the gantry moves in a circular path. Details on derivation of corrections are given in a previous paper [13]. Finally, (*EPID Sag*_*X*_)^*0*^ and (*EPID Sag*_*Y*_)^*0*^ are the EPID sag values at zero gantry angle.

### Gantry sag

To determine the values of gantry sag during rotation, positions of the centres of the four ball bearings attached to the head are determined in each image. The average of their positions in X and Y directions in each snapshot is used as a measure of the combined effect of EPID sag and gantry sag. Then, the gantry sag is simply determined at each angle by subtraction of the EPID sag (Equations 3 and 4).

3 $$ {\left( Gantry\ Sa{g}_X\right)}^{\theta }={\left\langle {a}_X,{b}_X,{c}_X,{d}_X\right\rangle}^{\theta }-{\left( EPID\ Sa{g}_X\right)}^{\theta } $$

4 $$ {\left( Gantry\ Sa{g}_Y\right)}^{\theta }={\left\langle {a}_Y,{b}_Y,{c}_Y,{d}_Y\right\rangle}^{\theta }-{\left( EPID\ Sa{g}_Y\right)}^{\theta } $$

where 〈a_X_, b_X_, c_X_, d_X_〉^θ^ and 〈a_Y_, b_Y_, c_Y_, d_Y_〉^θ^ are the averages of X and Y positions of the four ball bearings.

### Changes in SDD

The change in the source-to-detector distance as a result of gantry rotation is calculated using Equation (5). This effect is a result of both EPID sag and gantry wobble along the radiation beam direction.

5 $$ \varDelta {\mathrm{SDD}}^{\uptheta}={\mathrm{SDD}}^0\times \left(\left[\frac{{\left\langle {\mathrm{a}}_{\mathrm{X}},{\mathrm{c}}_{\mathrm{X}}\right\rangle}^{\uptheta} - {\left\langle {\mathrm{b}}_{\mathrm{X}},{\mathrm{d}}_{\mathrm{X}}\right\rangle}^{\uptheta}}{{\left\langle {\mathrm{a}}_{\mathrm{X}},{\mathrm{c}}_{\mathrm{X}}\right\rangle}^0 - {\left\langle {\mathrm{b}}_{\mathrm{X}},{\mathrm{d}}_{\mathrm{X}}\right\rangle}^0}\right]-1\right) $$

where SDD^0^ is the source-to-detector distance at zero gantry angle as read out from the DICOM header; 〈*a*_*X*_, *c*_*X*_〉^*θ*^ and 〈*b*_*X*_, *d*_*X*_〉^*θ*^ are the averaged positions of ball bearings (*a*) and (*c*), and (*b*) and (*d*), respectively at gantry angle θ.

### EPID and collimator skewness

The EPID and collimator skewness (yaw) during gantry rotation is determined using Equation (6) through a simple geometrical calculation based on the position of a pair of ball bearings ((*a*) and (*b*), or (*c*) and (*d*)) attached to the head at zero and θ gantry angles.

6 $$ {\uppsi}^{\uptheta}={ \tan}^{-1}\left(\frac{{\mathrm{b}}_{\mathrm{Y}}^{\uptheta}-{\mathrm{a}}_{\mathrm{Y}}^{\uptheta}}{{\mathrm{b}}_{\mathrm{X}}^{\uptheta}-{\mathrm{a}}_{\mathrm{X}}^{\uptheta}}\right)-{ \tan}^{-1}\left(\frac{{\mathrm{b}}_{\mathrm{Y}}^0-{\mathrm{a}}_{\mathrm{Y}}^0}{{\mathrm{b}}_{\mathrm{X}}^0-{\mathrm{a}}_{\mathrm{X}}^0}\right) $$

where Ψ^θ^ is the EPID and collimator skewness in degrees.

Skewness is considered as the rotation in EPID/collimator plane. The clockwise direction is taken as positive.

### EPID tilt

The EPID tilt in its plane during gantry rotation is derived from Equations (7) and (8) in the in-plane (pitch) and cross-plane (roll), respectively. Only the four ball bearings on the gantry head are used for the analysis.

7 $$ {\upvarphi}_Y^{\theta }={ \tan}^{-1}\left\{\frac{{\left[SD{D}^0\times \left(\left[\frac{a_X^{\theta }-{b}_X^{\theta }}{a_X^0-{b}_X^0}\right]-1\right)\right]}_G - {\left[SD{D}^0\times \left(\left[\frac{c_X^{\theta }-{d}_X^{\theta }}{c_X^0-{d}_X^0}\right]-1\right)\right]}_T}{{\left\langle {\mathrm{a}}_{\mathrm{Y}},{\mathrm{b}}_{\mathrm{Y}}\right\rangle}^{\uptheta}-{\left\langle {\mathrm{c}}_{\mathrm{Y}},{\mathrm{d}}_{\mathrm{Y}}\right\rangle}^{\uptheta}}\right\} $$

8 $$ {\upvarphi}_X^{\theta }={ \tan}^{-1}\left\{\frac{{\left[SD{D}^0\times \left(\left[\frac{a_Y^{\theta }-{b}_Y^{\theta }}{a_Y^0-{b}_Y^0}\right]-1\right)\right]}_L - {\left[SD{D}^0\times \left(\left[\frac{c_Y^{\theta }-{d}_Y^{\theta }}{c_Y^0-{d}_Y^0}\right]-1\right)\right]}_R}{{\left\langle {\mathrm{a}}_{\mathrm{X}},{\mathrm{b}}_{\mathrm{X}}\right\rangle}^{\uptheta}-{\left\langle {\mathrm{c}}_{\mathrm{X}},{\mathrm{d}}_{\mathrm{X}}\right\rangle}^{\uptheta}}\right\} $$

The indices G, T, L and R denote the gun, target, left and right directions.

### Sag in the MLC carriages

The sag in the MLC carriages corresponding to each gantry angle in the G, T, L and R directions in the in-plane and cross-plane directions are quantified using Equations (9) to (12).

9 $$ ML{C}_{Sag,\ L}^{\theta }={\left\langle {a}_X,{b}_X,{c}_X,{d}_X\right\rangle}^{\theta }-{L}_{Edge}^{\theta } $$

10 $$ ML{C}_{Sag,R}^{\theta }={R}_{Edge}^{\theta }-{\left\langle {a}_X,{b}_X,{c}_X,{d}_X\right\rangle}^{\theta } $$

11 $$ ML{C}_{Sag,G}^{\theta }={\left\langle {a}_Y,{b}_Y,{c}_Y,{d}_Y\right\rangle}^{\theta }-{G}_{Edge}^{\theta } $$

12 $$ ML{C}_{Sag,T}^{\theta }={T}_{Edge}^{\theta }-{\left\langle {a}_Y,{b}_Y,{c}_Y,{d}_Y\right\rangle}^{\theta } $$

where ${L}_{Edge}^{\theta }$, *etc*. represent the positions of four field edges at each gantry angle. ${L}_{Edge}^{\theta }$ and ${R}_{Edge}^{\theta }$, are based on the averaged leaf positions in each bank.

## Results

Results of investigations on EPID sag, gantry sag, changes in SDD, the skewness of EPID and collimator, EPID tilt, and the sag in MLC carriages are given separately in this section. Each set of results for gantry rotation in CW direction and zero collimator angle is presented in graphs and every point is given as the average ±1SD. Each graph contains more than 950 points.

### EPID sag

**Figure 2:**
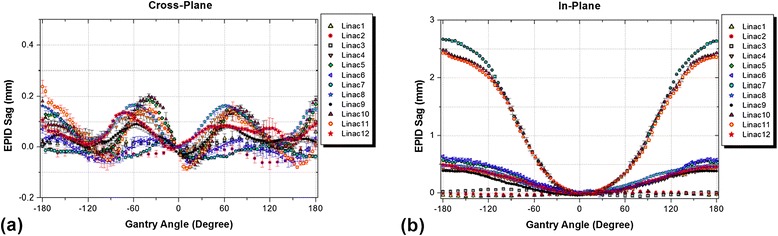
**Comparison of EPID sag measurement (mean ±1SD) results for the tested linacs in: (a) cross-plane, and (b) in-plane directions.**

Results of EPID sag measurements over all linacs are given in Figure 2.

The values of EPID sag were less than 0.3 mm in the cross-plane direction in all linacs, while larger deviations were observed in the in-plane direction. Three of the linacs had EPID sag values larger than 2.5 mm while the accepted criterion for non-stereotactic linacs is 2 mm, based on the AAPM TG 142 report [8]. The three linacs with smallest EPID sag values were all TrueBeams.

### Gantry sag

**Figure 3:**
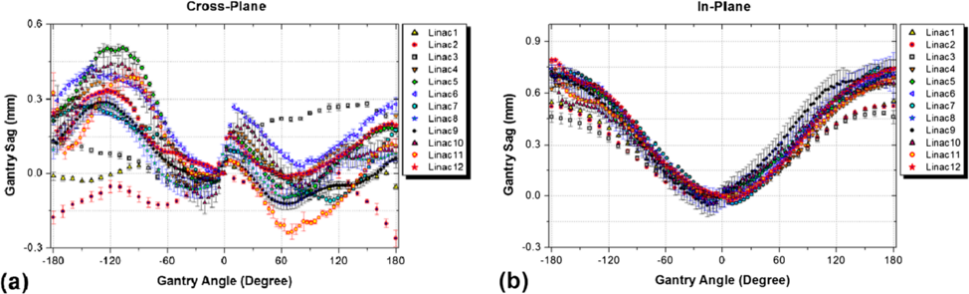
**Comparison of gantry sag measurement results for the tested linacs in: (a) cross-plane, and (b) in-plane directions.**

Measurement results of gantry sag at various gantry angles for all linacs are given in Figure 3.

As shown in Figure 3, more differences were observed among gantry sag patterns in the cross-plane direction compared to the in-plane. However, the measured values for all linacs in both directions were less than the 1 mm acceptance criterion [8].

### Changes in SDD

**Figure 4:**
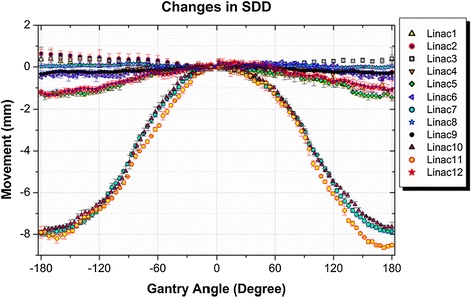
**Comparison of the results of changes in SDD for the tested linacs.**

Results of the measured changes in the source-to-detector distance in the beam direction are shown in Figure 4.

Figure 4 shows that the change in SDD in most of the tested linacs was less than the 5 mm accepted criterion [8]. However, the largest change in the SDD was 8.65 mm which results in 0.58% image magnification and corresponds to 1.15% change in dose when the EPID is used for absolute dosimetry. The three linacs with larger SDD variations were in service at the same centre.

### EPID and collimator skewness

**Figure 5:**
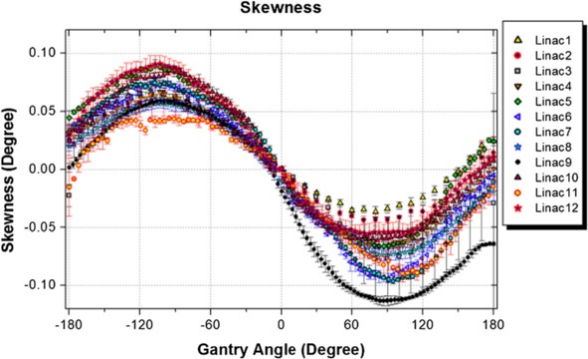
**Comparison of the measured skewness in EPID and collimator for the tested linacs.**

Results of measurements of the EPID and collimator skewness for all linacs are compared in Figure 5. The patterns of EPID and collimator skewness were similar and their values were below 0.2 degrees for all tested linacs.

### EPID tilt

**Figure 6:**
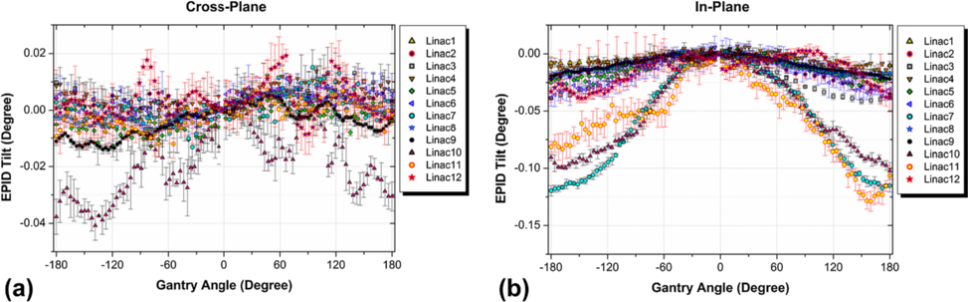
**Comparison of EPID tilt measurement results for the tested linacs in: (a) cross-plane, and (b) in-plane directions.**

Results of the EPID tilt measurements for all linacs are given in Figure 6. The detected EPID tilt values were negligible for all tested linacs. The scale of graphs in the figure indicates the precision of the algorithm.

### Sag in the MLC carriages

**Figure 7:**
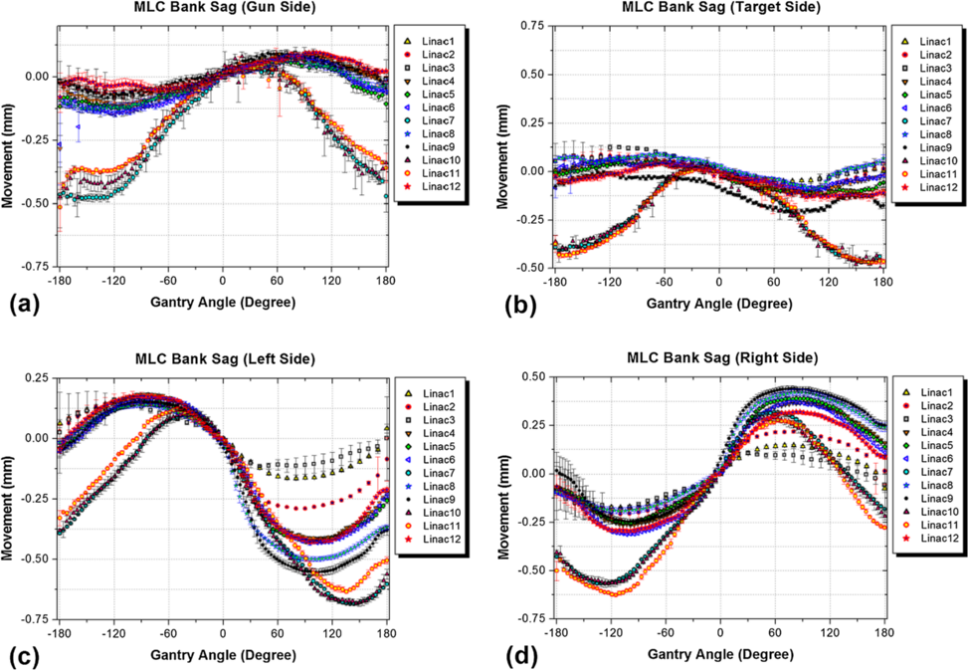
**Comparison of the measured sag values in leaf bank assemblies of the tested linacs for: (a) gun side, (b) target side, (c) left side, and (d) right side.** Each data point represents the average over three sets of measurements made at zero collimator angle.

Figure 7 shows the measured sag patterns in the MLC carriages of the tested linacs in four directions.

The range of sag in the leaf bank assemblies -which produces a systematic error- was less than 1 mm in all directions over all tested linacs. Although the acceptance limit for deviations in MLC positioning is within 1 mm [8], Rangel and Dunscombe have shown that the systematic error in leaf positions should be limited to 0.3 mm for acceptable clinical outcomes [16].

### Summary of results

A statistical summary of the results over all linacs is given in Tables 2 and 3 for each of the investigated components. Table 2 includes the average and largest range of deviations and the worst reproducibility for each component. The range of variations is considered as the difference between maximum and minimum data values for each linac. The worst reproducibility belongs to the linac with the largest standard deviation of results amongst the tested machines (over three sets of measurements). The linac number corresponding to each of the above is also provided. Data are produced for both the in-plane and cross-plane directions where applicable. Only the largest deviations are listed in the table.

**Table 2:** **Statistical comparison of the tested linacs for the average and largest range of deviations, and the maximum standard deviations of the results (worst reproducibility) over each of the tested components**

		**Average range**	**Largest range**	**Worst reproducibility**
**EPID Sag (mm)**	Cross-plane	0.17	0.32 (L#11)	0.04 (L#11)
	In-plane	1.00	2.68 (L#7)	0.08 (L#10)
**Gantry Sag (mm)**	Cross-plane	0.42	0.63 (L#11)	0.10 (L#10)
	In-plane	0.72	0.82 (L#12)	0.15 (L#8)
**SDD (mm)**		3.57	8.65 (L#11)	1.15 (L#2)
**EPID and Collimator Skew (degrees)**		0.15	0.17 (L#9)	0.09 (L#3)
**EPID Tilt (degrees)**	Cross-plane	0.02	0.04 (L#10)	0.01 (L#7)
	In-plane	0.06	0.13 (L#11)	0.05 (L#11)
**MLC Sag (mm)**	Left	0.62	0.82 (L#8)	0.17 (L#3)
	Right	0.68	0.99 (L#8)	0.15 (L#3)
	Gun	0.34	0.57 (L#11)	0.16 (L#6)
	Target	0.32	0.61 (L#10)	0.10 (L#3)

Values are in mm except for the EPID tilt and skew which are given in degrees. The largest measured values are listed and their corresponding linac numbers are given in parentheses.

**Table 3:** **Statistical comparison of the tested linacs using the RMSD of measured data in CW and CCW directions, and using 0 and 90 degree collimators**

		**CW vs. CCW**		**Col 0 vs. Col90**
		**Average RMSD**	**Maximum RMSD**	**Maximum RMSD**
**EPID Sag (mm)**	Cross-plane	0.07	0.44 (L#7)	0.00 (L#12)
	In-plane	0.22	1.61 (L#7)	0.01 (L#7)
**Gantry Sag (mm)**	Cross-plane	0.11	0.26 (L#6)	0.07 (L#2)
	In-plane	0.04	0.12 (L#8)	0.02 (L#9)
**SDD (mm)**		0.60	0.81 (L#7)	0.00 (L#7)
**EPID and Collimator Skew (degrees)**		0.16	1.62 (L#7)	0.01 (L#7)
**EPID Tilt (degrees)**	Cross-plane	0.06	0.07 (L#7)	0.01 (L#7)
	In-plane	0.01	0.08 (L#7)	0.01 (L#12)
**MLC Sag (mm)**	Left	0.19	0.30 (L#9)	0.38 (L#7)
	Right	0.21	0.54 (L#7)	0.38 (L#7)
	Gun	0.07	0.18 (L#7)	0.41 (L#3)
	Target	0.06	0.19 (L#3)	0.47 (L#3)

Values are in mm except for the EPID tilt and skew which are given in degrees. The largest deviations are listed and their corresponding linac numbers are given in parentheses.

Table 3 represents a comparison between measurements performed with CW and CCW gantry rotations and with collimator setting at 0 and 90 degrees across all linacs. Comparison is made using the root mean square deviation (RMSD) of results for each set of measurements.

**Figure 8:**
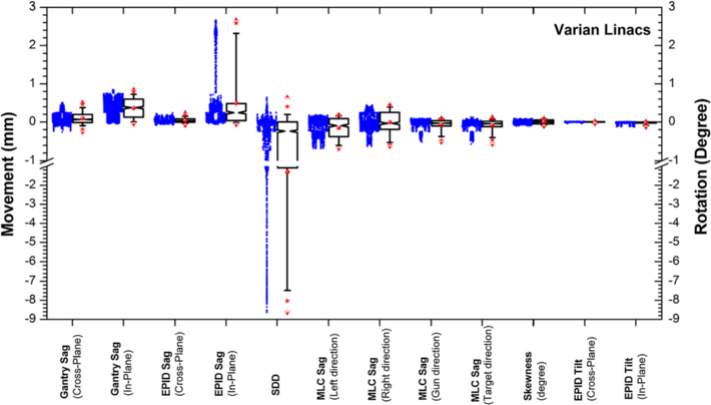
**A box plot for the data from tested linacs at zero collimator angle and CW gantry rotations.** All values are in mm except for the skewness and EPID tilt which are in degrees. The triangles represent the maximum and minimum of data points, the stars show 99% and 1% confidence intervals (CI) of the medians, the whiskers represent 95% and 5% CI of each median, and the upper and lower ends of the box are at 75% and 25% CI of the median. The actual distribution of measured data points are shown on the left side of each box.

A box plot containing the results of measured data with CW gantry rotations are given in Figure 8. It provides a descriptive statistical illustration that summarizes the results by showing the spread of points over the whole sets of measured data across all linacs.

The acquired data were further analysed to test if a relationship could be found between the linac performance with their model and the number of years they were in service. The correlation coefficient between the average discrepancies for each linac model (Clinac, Trilogy, TrueBeam) and the average number of years in service for linacs of the same model was found to be 0.9. This means there is a strong linear relationship between the linac performance and its age in all models.

## Discussion

A comprehensive study was performed on a range of Varian linacs to investigate the mechanical stability of their gantries, MLC leaf bank assemblies, and MV imagers during arc delivery. All of these linac components are affected by gravitational force during gantry rotation due to their structural imperfections. Information on the impact of rotation on these systems will assist in delivery of more accurate treatments by improving pre- and post-treatment verification of complex treatments, and linac QA processes.

In this study, a simple measurement method is proposed to simultaneously quantify the gantry, leaf bank, and EPID movements during an entire gantry rotation. A simple phantom was designed with five metallic markers in the beam, and a large amount of information on the system characteristics were extracted from a set of EPID images using in-house developed fast, robust and accurate software. The software automatically analyses all of the EPID images acquired at different gantry angles and calculates their geometric parameters.

Results of EPID sag measurements in all linacs showed that on average, the EPID movements had a range of 0.2 mm in the cross-plane and 1.0 mm in the in-plane directions. EPID sag in the in-plane direction was expected to be larger since the EPID can move more freely in this direction due to its structure. Three of the linacs (No. 7, 10, and 11) had out-of-range values for EPID sag in the in-plane direction. Interestingly, they were all operating at the same centre; therefore, after discussing the matter with the department the observed issue was attributed to the lack of routine service and maintenance of the support arm components. Three other linacs (all TrueBeams) had much smaller sag values in the in-plane direction compared to other machines. The first reason is that in TrueBeam linacs the supervisor module provides monitoring of sub-systems every 10 ms to control the function of several components including the detector positioning unit. This has shown to result in improved clinical delivery for TrueBeams over Clinacs [17] which is consistent with the findings of this study. The position of each axis of the supporting arm (shoulder, elbow and wrist joints) is measured by at least two sensors that connect directly to the arm’s axis by gears and/or belt. This provides improved EPID positioning accuracy in TrueBeam systems [18]. The second reason for improved EPID sag results in TrueBeam linacs in this study is that all of them were calibrated using an IsoCal phantom on a monthly basis. The IsoCal phantom has recently been introduced by Varian Medical Systems as a tool for geometric calibration of imager arms and for tuning its alignment in the TrueBeam model. It includes 16 tungsten carbide ball bearings arranged in a certain projection pattern unique for each gantry angle and an aluminium partial transmission plate attached to the gantry head with a hole in the middle [19,20]. However, this system has some limitations such as the need for aligning the phantom at the isocentre with an accuracy of 5 mm, dependence on the accuracy of room lasers for phantom alignment, need for accurate manufacture of the phantom, and not being able to cover the whole 360 degree rotation of gantry. In addition, the shadow of the aluminium plate can introduce errors in the analysis, and if the variation of arm trajectories is not smooth the accuracy of algorithm will be affected [18]. The method suggested in this study has none of these restrictions. The phantom does not need to be accurately positioned. It should only be fixed firmly so that it does not move during gantry rotation. In this method, information is provided not only on EPID sag, but also on several other mechanical characteristics of the linac simultaneously, and the software needs to be run only once to analyse all the data.

If a Varian EPID shows large values of sag, it would be advisable to check the tightness of fasteners since nuts or bolts could loosen due to vibration. Gear belts, linear drive and lateral guiding rails may need to be checked too.

The average range of gantry sag values was 0.7 mm in the in-plane and 0.4 mm in the cross-plane directions (Table 2). These values were consistent with previous reports on Varian machines [10,13]. A discontinuity was observed around zero gantry angle in the cross-plane direction for all machines. This effect was similarly observed in previous studies [10,13,21].

If large gantry sag values are detected in a Varian linac, it may indicate tension in the gantry chain. It may be necessary to check the gantry bearing and the collimator rotation bearing.

The change in source-to-detector distance during gantry rotation excluding the three linacs with maintenance issues was 1.73 mm on average. Three of the linacs showed changes in SDD outside the accepted criterion. These were the same three machines that had the largest EPID sag values in the in-plane direction. This is comparable with our previous report on Varian machines equipped with E-type arms [13]; however, older Varian linacs with R-type arms have shown changes in SDD up to 13 mm [4,22]. The values found for EPID and collimator skewness, and EPID tilt were reported for the first time and were found to be less than 1 degree. Values for EPID tilt in the in-plane direction were larger due to the presence of a joint in the arm structure (known as the wrist joint).

In Varian linacs, the MLC leaves and drive motors, and the MLC controller system are mounted in carriage boxes (more than 36 kg each) on each side (left and right). These boxes can move under the influence of gravity; therefore, although displacements of each individual leaf can be accurately detected, the MLC controller system is unable to recognize the sag in the MLC leaf bank carriages.

The average sag in the leaf carriages of the tested linacs were about 0.3 mm in the gun and target directions, and about 0.7 mm in the left and right directions. The difference was expected based on the structure of the MLC system and the direction of gravitational force. The largest range of sag did not exceed 1 mm in any direction. It must be noted that in Figure 7(c) and (d), the marker positions remain unchanged since they are fixed to the gantry head. At −90°, the distance between the marker and left MLC bank is at its maximum since the gravity force is strongest on the left bank enforcing it to move to its maximum possible shift. The distance gradually decreases and reaches its minimum at the 90° gantry angle. The pattern is in the opposite direction for the right bank. More detail is given in a previous paper [13]. The three linacs which had the maximum deviations in previous components showed different patterns in MLC carriage behaviour in arc compared to other machines. It worth mentioning that the sag in both the gantry and the MLC system affect the patient and must be added for clinical considerations.

Based on the results listed in Table 2, the results were highly reproducible and therefore, the machine behaviour was consistent and the method is solid. The data in Table 3 showed that the average RMSD was small between CW and CCW gantry rotations. The largest difference in mm was observed for EPID sag in the in-plane direction for linac number 7. The department has been informed and maintenance is recommended. Collimator rotation from 0 to 90 degrees had no major effect on the mechanical behaviour of machine components during arc. The effect on MLC carriage, though small, was larger than other components since the rotation of collimator changed the parts that were under the largest influence of gravity.

The method introduced in this study can be extended to kV imagers (source and detector) since it is essential to characterize the kV system for accurate patient positioning.

## Conclusion

With the introduction of complex technologies in modern radiotherapy, more accurate and efficient methods are required to ensure correct delivery of treatments. In this work, a large amount of information on characteristics of Varian linac components during arc deliveries were provided using EPID images acquired with five metallic markers in the beam. A fast and accurate software package was developed for the analysis of images and several linacs of different models were investigated and compared with the reports in the literature.

## Abbreviations

EPID: Electronic portal imaging device
IMRT: Intensity modulated radiation therapy
VMAT: Volumetric modulated radiation therapy
QA: Quality assurance
SDD: Source-to-detector distance
DICOM: Digital imaging and communications in medicine
MU: Monitor unit
RMSD: Root mean square deviation
CW: Clockwise
CCW: Counter clockwise
CI: Confidence interval
